# Transactions of the American Institute of Homœopathy

**Published:** 1887-03

**Authors:** 


Transactions of the American Institute of Homœopathy. Thirty-ninth Session, held at Saratoga Springs, in June, 1886. Edited by the Secretary, J. C. Burgher, M. D. Pittsburg: Stevenson & Foster. 1886.

This large volume, which makes its yearly appearance, is now on hand. A great number of articles by different members is published, but we have not space even to enumerate them. W. M. J.

